# Association of SARS-CoV-2 Infection During Controlled Ovarian Stimulation With Oocyte- and Embryo-Related Outcomes

**DOI:** 10.1001/jamanetworkopen.2023.23219

**Published:** 2023-07-13

**Authors:** Fen Tian, Saijiao Li, Ning Li, Hao Zhao, Man Luo, Jing Zhang, Zenghui Mao, Qianjie Zhang, Rong Li, Tingting Tang, Cuilian Zhang, Yanping Li, Shaodi Zhang, Jing Zhao

**Affiliations:** 1 Reproductive Medicine Center, Xiangya Hospital, Central South University, Changsha, Hunan, China; 2 Clinical Research Center for Women’s Reproductive Health in Hunan Province, Changsha, Hunan, China; 3 Reproductive Medicine Center, Renmin Hospital of Wuhan University, Wuhan, China; 4 Reproductive Medicine Center, The Third Affiliated Hospital of Guangxi Medical University, Nanning, Guangxi, China; 5 Reproductive Medicine Center, Hunan Provincial Maternal and Child Health Hospital, Changsha, Hunan, China; 6 Reproductive Medicine Center, The First Affiliated Hospital of University of South China, Hengyang, Hunan, China; 7 Reproductive Medicine Center, Changsha Hospital for Maternal & Child Health Care, Changsha, Hunan, China; 8 Reproductive Medicine Center, Henan Provincial People’s Hospital, Zhengzhou, China

## Abstract

**Importance:**

SARS-CoV-2 infection has had significant effects on the health of people worldwide. Whether SARS-CoV-2 infection during controlled ovarian stimulation (COS) is associated with laboratory outcomes in assisted reproductive technology remains unclear.

**Objective:**

To investigate the association between SARS-CoV-2 infection during COS with oocyte- and embryo-related outcomes.

**Design, Setting, and Participants:**

A multicenter cohort study was conducted of couples undergoing assisted reproductive technology treatments in 7 reproductive centers in 4 provinces in China from October 1, 2022, to December 31, 2022. All couples received nucleic acid testing for SARS-CoV-2 during COS. The SARS-CoV-2–positive group included couples in which either partner was infected with SARS-CoV-2. The SARS-CoV-2–negative group comprised couples without infection.

**Exposure:**

In the SARS-CoV-2–positive group, either partner was infected with SARS-CoV-2 during COS, defined as a positive test result for the SARS-CoV-2 antigen.

**Main Outcomes and Measures:**

Primary outcomes were the available embryo and blastocyst and top-quality embryo and blastocyst rates. Secondary outcomes were the number of oocytes retrieved, the mature oocyte rate, normal fertilization (2 pronuclei observed on day 1 after insemination [2PN]), oocyte degeneration, 2PN cleavage, and blastocyst formation rates.

**Results:**

A total of 585 heterosexual couples with infertility participated in the study (median [IQR] age for female partners, 33 [30-37] years), with 135 couples in the SARS-CoV-2–positive group and 450 in the SARS-CoV-2–negative group. The characteristics of the groups were similar. The SARS-CoV-2–positive group had a significantly lower top-quality embryo rate (odds ratio [OR], 0.83; 95% CI, 0.71-0.96), top-quality blastocyst rate (OR, 0.59; 95% CI, 0.45-0.77), available blastocyst rate (OR, 0.70; 95% CI, 0.59-0.82), and blastocyst formation rate (OR, 0.61; 95% CI, 0.52-0.71) than the SARS-CoV-2–negative group. Analysis of the associations of infection by sex showed that the female positive group had impaired oocyte and embryo quality regarding mature oocyte rate, 2PN cleavage rate, top-quality embryo rate, blastocyst formation rate, available blastocyst rate, and top-quality blastocyst rate compared with the SARS-CoV-2–negative group. Compared with the SARS-CoV-2–negative group, the male positive group and the group of couples with both positive partners had significantly decreased available blastocyst rate, top-quality blastocyst rate, and blastocyst formation rate compared with the SARS-CoV-2 negative group.

**Conclusions and Relevance:**

In this cohort study, SARS-CoV-2 infection during COS was negatively associated with embryo and blastocyst quality. Reproductive physicians should be more attentive to patients with SARS-CoV-2 infection during COS and should give couples who have been infected adequate counseling.

## Key Points

**Question:**

Is SARS-CoV-2 infection during controlled ovarian stimulation (COS) associated with oocyte- and embryo-related outcomes?

**Findings:**

This multicenter cohort study of 585 couples with infertility found that couples with SARS-CoV-2 infection had significantly lower top-quality embryo and blastocyst, blastocyst formation, and available blastocyst rates than couples without infection. SARS-CoV-2 infection during COS was associated with embryo and blastocyst quality.

**Meaning:**

This study suggests that reproductive physicians should pay attention to patients with SARS-CoV-2 infection during COS and should give these couples adequate counseling.

## Teaser

This cohort study of couples with infertility evaluates whether SARS-CoV-2 infection during controlled ovarian stimulation is associated with oocyte- and embryo-related outcomes.

## Introduction

In the past 3 years, SARS-CoV-2 infection and COVID-19 have had significant effects on the health of people worldwide. In China, the control of COVID-19 has been relaxed since December 2022, and the number of couples infected with SARS-CoV-2 during assisted reproductive technology (ART) treatment increased sharply during that period. Angiotensin-converting enzyme 2 (ACE2) and transmembrane protease serine subtype 2 (TMPRSS2) are expressed in the reproduction system in both males and females^1^; therefore, reproductive function is theoretically vulnerable to SARS-CoV-2 infection, and many studies^2,3^ have explored the possible effect of SARS-CoV-2 infection on the human reproductive system. A recent review^4^ summarized published data and clarified the effect of SARS-CoV-2 and COVID-19 on human fertility and assisted reproduction.

However, the association between SARS-CoV-2 infection and ART treatment outcomes remains unclear. Several studies^5,6^ have assessed the association between a previous SARS-CoV-2 infection in infertile couples undergoing ART and treatment outcomes. Few studies have reported ART outcomes in couples with infertility infected with COVID-19 during ART. One study^7^ did not detect viral RNA in follicular fluid, cumulus cells, or endometrial tissue samples in 16 SARS-CoV-2–infected women and indicated that the fertilization rate and embryo development were reassuring. Another study^8^ showed a diminished oocyte utilization rate of 2 pronuclear (2PN) zygotes and good-quality embryos in women infected within 7 days before oocyte retrieval. Existing studies, with small sample sizes, have yet to reach a consensus.

To date, the association of SARS-CoV-2 infection with oocytes and embryos during controlled ovarian stimulation (COS) of ART has yet to be elucidated. Therefore, the current study aimed to evaluate whether SARS-CoV-2 infection during COS was negatively associated with oocyte- and embryo-related outcomes of ART.

## Methods

### Study Population and Design

We performed a multicenter, retrospective cohort study of couples in which the woman was undergoing in vitro fertilization (IVF) or intracytoplasmic sperm injection (ICSI) treatment between October 1, 2022, and December 31, 2022, from 7 reproductive centers in 4 provinces across China. The current study was approved by the ethics committee of Xiangya Hospital, Central South University, which determined that the study was exempt from informed consent requirements owing to its retrospective design. This study followed the Strengthening the Reporting of Observational Studies in Epidemiology (STROBE) reporting guideline for observational studies.

The inclusion criteria were as follows: (1) undergoing COS and IVF or ICSI and (2) testing for SARS-CoV-2 during COS treatment. Couples who met the following criteria were excluded: (1) undergoing oocyte or sperm donation cycles, (2) undergoing oocyte freezing cycles, (3) failure to obtain oocytes, and (4) lost to follow-up. All included couples were divided into 2 groups according to nucleic test result for SARS-CoV-2: the SARS-CoV-2–positive group (1 partner or both partners infected with SARS-CoV-2) and the SARS-CoV-2–negative group (both uninfected with SARS-CoV-2).

To clarify the association between infection and oocyte- and embryo-related outcomes by sex, the SARS-CoV-2–positive group was further subdivided into 3 groups: the female SARS-CoV-2–positive group (only the female partner was infected with SARS-CoV-2), male SARS-CoV-2–positive group (only the male partner was infected with SARS-CoV-2), and both-positive group (both individuals in the couple were infected with SARS-CoV-2). To eliminate the possible confounding factors resulting from different reproductive centers, the same proportion of sample size of the SARS-CoV-2–positive group vs the SARS-CoV-2–negative group was ensured for each reproductive center.

### Ovarian Stimulation and Embryo Culture

The COS protocols were performed as previously described.^9,10^ The COS protocols were categorized into gonadotropin-releasing hormone (GnRH) agonist, GnRH antagonist, and other protocols, including mild stimulation and progestin-primed ovarian stimulation protocols. Follicular development was monitored using transvaginal ultrasonography. When 2 or 3 follicles with diameters of 17 mm or greater were observed, 6000 to 10 000 IU of human chorionic gonadotropin was administered and oocyte retrieval was performed 36 to 38 hours later. Either IVF or ICSI was performed according to sperm quality. Fresh embryos or blastocyst transfers were canceled and cryopreserved.

### Outcome Assessments

Demographic and COS cycle characteristics and oocyte and embryo parameters were recorded. The primary outcomes were the available embryo and blastocyst rates and top-quality embryo and blastocyst rates. The secondary outcomes were the number of oocytes retrieved, the mature oocyte rate, normal fertilization (2PN), oocyte degeneration, 2PN cleavage, and blastocyst formation rates. Laboratory indicators were calculated as follows: mature oocyte rate = number of metaphase 2 oocytes/number of oocytes retrieved (ICSI cycles); oocyte degeneration rate = number of degenerated oocyte/the number of metaphase 2 oocytes (ICSI cycles); normal fertilization rate (IVF cycles) = number of 2PN zygotes / oocytes retrieved; normal fertilization rate (ICSI cycles) = number of 2PN zygotes / number of metaphase 2 oocytes; cleavage rate = number of cleavage embryos from 2PN / number of 2PN zygotes; available embryo rate = number of embryos for cryopreservation / number of cleavage embryos from 2PN; top-quality embryo rate = number of top-quality embryos / number of 2PN cleavage embryos; blastocyst formation rate = number of blastocysts / number of 2PN cleavage embryos; available blastocysts rate = number of blastocysts for cryopreservation / number of 2PN cleavage embryos; and top-quality blastocyst rate = number of the top-quality blastocysts / number of 2PN cleavage embryos. Embryo grading of each reproductive center was based on the Istanbul consensus workshop parameters.^11^

### Statistical Analysis

All data were analyzed using SPSS software, version 26.0 (IBM Corp). Normality was tested using the Shapiro-Wilk test. Continuous variables are expressed as means (SDs) for normally distributed data and as medians (IQRs) for nonnormally distributed data. Two-tailed *t* tests or Mann-Whitney *U* tests were performed for statistical analysis. Categorical data were described as number (percentage) and analyzed by the χ^2^ test or Fisher exact test. Multiple linear regression models were used to adjust for confounding factors on outcomes with significant difference. A 2-sided *P* < .05 was considered to indicate statistical significance. The Bonferroni method with adjusted significance threshold (.017 [0.05/3]) was applied to handle the potential for type I error.

## Results

A total of 585 heterosexual couples with infertility (median [IQR] age, 33 [30-37] years for female partners) were included in the current study, with 135 in the SARS-CoV-2–positive group and 450 in the SARS-CoV-2–negative group. The patients’ clinical characteristics and details of their IVF or ICSI cycles are given in Table 1. The median (IQR) body mass index (BMI; calculated as weight in kilograms divided by height in meters squared) for the female partners was 22.1 (20.4-24.4). The median (IQR) duration of infertility was 2 (1-4) years, and 314 couples (53.7%) had secondary infertility. The major infertility factor was a female factor in 341 couples (58.3%). The GnRH antagonist COS protocol was used in 296 (50.6%) of the infertile couples. No significant differences were found in female age, BMI, infertility type, infertility factors, and COS protocols between the SARS-CoV-2–positive and SARS-CoV-2–negative groups, except for infertility duration, which was longer in the SARS-CoV-2–negative group than the SARS-CoV-2–positive group (median [IQR], 3.0 [1.0-4.4] years vs 2.0 [1.0-4.0] years; *P* = .03). In general, baseline characteristics were comparable between 2 groups.

**Table 1.** Baseline Characteristics of SARS-CoV-2–Positive and SARS-CoV-2–Negative Groups^a^

Characteristic	Total (N = 585)	SARS-CoV-2 negative (n = 450)	SARS-CoV-2 positive (n = 135)
Female partner’s age, median (IQR), y	33 (30-37)	33 (30-37)	33 (28-37)
Female partner’s BMI, median (IQR)	22.1 (20.4-24.4)	22.0 (20.3-24.4)	22.6 (20.7-24.8)
No. of treatment cycles, median (IQR), No.	1 (1-2)	1 (1-2)	1 (1-2)
Infertility duration, median (IQR), y	2.0 (1.0-4.0)	3.0 (1.0-4.4)	2.0 (1.0-4.0)
Infertility type			
Primary	271 (46.3)	205 (45.6)	66 (48.9)
Secondary	314 (53.7)	245 (54.4)	69 (51.1)
Infertility factor			
Female factor	341 (58.3)	260 (57.8)	81 (60.0)
Male factor	41 (7.0)	35 (7.8)	6 (4.4)
Both female and male factors	181 (30.9)	138 (30.7)	43 (31.9)
Unexplained	22 (3.8)	17 (3.8)	5 (3.7)
COS protocols			
GnRH agonist	167 (28.5)	131 (29.1)	36 (26.7)
GnRH antagonist	296 (50.6)	230 (51.1)	66 (48.9)
Others^b^	122 (20.9)	89 (19.8)	33 (24.4)

Abbreviations: BMI, body mass index (calculated as weight in kilograms divided by height in meters squared); COS, controlled ovarian stimulation; GnRH, gonadotropin-releasing hormone.

^a^ Data are presented as number (percentage) of study couples unless otherwise indicated.

^b^ Others, including mild stimulation and luteal-phase stimulation protocols.

As shown in Table 2, the ovarian reserve and ovarian response characteristics in terms of basal follicle-stimulating hormone, antral follicle count, antimüllerian hormone, duration of COS, dosage of gonadotropin, estradiol, progesterone, and luteinizing hormone on the trigger day and the number of oocytes retrieved were not significantly different between the 2 groups. No significant differences were observed in oocyte- and oocyte-related outcomes in the fertilization methods selection, mature oocyte rate (in ICSI fertilization), oocyte degeneration rate, 2PN rate, 2PN cleavage rate, and available embryo rate between the 2 groups. The top-quality embryo rate (434 of 918 [47.3%] vs 1557 of 2990 [52.1%]; odds ratio [OR], 0.83; 95% CI, 0.71-0.96; *P* = .01), blastocyst formation rate (306 of 918 [33.3%] vs 1353 of 2990 [45.3%]; OR, 0.61; 95% CI, 0.52-0.71; *P* < .001), available blastocyst rate (259 of 918 [28.2%] vs 1070 of 2990 [35.8%]; OR, 0.70; 95% CI, 0.59-0.82; *P* < .001) and top-quality blastocyst rate (65 of 918 [7.1%] vs 343 of 2990 [11.5%]; OR, 0.59; 95% CI, 0.45-0.77; *P* < .001) in the SARS-CoV-2–positive group were significantly lower than those in the SARS-CoV-2–negative group (Table 3).

**Table 2.** Fertility Evaluation and COS Results for the SARS-CoV-2–Positive and SARS-CoV-2–Negative Groups

Variable	Median (IQR)		Median difference (95% CI)	*P* value
	SARS-CoV-2 negative (n = 450)	SARS-CoV-2 positive (n = 135)		
Basal FSH, mIU/mL	6.7 (5.6 to 8.5)	6.5 (5.3 to 7.8)	0.30 (−0.12 to 0.72)	.15
AMH, ng/mL	2.5 (1.3 to 3.9)	3.7 (1.3 to 4.5)	−0.14 (−0.52 to 0.23)	.44
AFC, No.	12 (7 to 18)	12 (7 to 19)	0 (−2 to 1)	.77
Duration of COS, d	9 (8 to 11)	9 (8 to 11)	0 (0 to 0)	.78
Dosage of gonadotropin, IU	2093.8 (1500.0 to 2614.9)	1975.0 (1475.0 to 2625.0)	75 (−75 to 225)	.34
Hormone levels on trigger day				
Estradiol, pg/mL	2036.0 (1314.5 to 3129.0)	1878.0 (1077.4 to 3000.0)	162.00 (−79.39 to 407.60)	.18
Progesterone, ng/mL	0.8 (0.6 to 1.2)	0.7 (0.5 to 1.2)	0.06 (−0.03 to 0.14)	.22
Luteinizing hormone, IU/L	2.2 (1.2 to 3.9)	2.2 (1.0 to 3.9)	0.08 (−0.24 to 0.39)	.63
Oocytes retrieved, No.	10 (6 to 15)	10 (6 to 15)	0 (−1 to 1)	.95

Abbreviations: AFC, antral follicle count; AMH, antimüllerian hormone; COS, controlled ovarian stimulation; FSH, follicle-stimulating hormone.

SI conversion factors: To convert FSH to international units per liter, multiply by 1; estradiol to picomoles per liter, multiply by 3.671; progestin to international units per liter, multiply by 1.

**Table 3.** Laboratory Outcomes of the SARS-CoV-2–Positive and SARS-CoV-2–Negative Groups

Variable	No./total No. (%)		OR (95% CI)	*P* value^a^
	SARS-CoV-2 negative (n = 450)	SARS-CoV-2 positive (n = 135)		
Fertilization methods				
IVF	318 (70.7)	101 (74.8)	NA	.64
ICSI	113 (25.1)	29 (21.5)	NA	
IVF and ICSI	19 (4.2)	5 (3.7)	NA	
Mature oocyte rate (ICSI)	981/1228 (79.9)	297/374 (79.4)	0.97 (0.73 to 1.29)	.84
Oocyte degeneration rate (ICSI)	75/981 (7.6)	14/297 (4.7)	0.60 (0.33 to 1.07)	.08
2PN rate				
IVF	2155/3476 (62.0)	687/1095 (62.7)	1.03 (0.90 to 1.19)	.66
ICSI	735/981 (74.9)	226/297 (76.1)	1.06 (0.79 to 1.44)	.68
2PN cleavage rate	2990/3059 (97.7)	918/947 (96.9)	0.73 (0.47 to 1.13)	.16
Top-quality embryo rate	1557/2990 (52.1)	434/918 (47.3)	0.83 (0.71 to 0.96)	.01
Available embryo rate	2448/2990 (81.9)	741/918 (80.7)	0.93 (0.77 to 1.12)	.43
Blastocyst formation rate	1353/2990 (45.3)	306/918 (33.3)	0.61 (0.52 to 0.71)	<.001
Available blastocyst rate	1070/2990 (35.8)	259/918 (28.2)	0.70 (0.59 to 0.82)	<.001
Top-quality blastocyst rate	343/2990 (11.5)	65/918 (7.1)	0.59 (0.45 to 0.77)	<.001

Abbreviations: 2PN, 2 pronuclei; ICSI, intracytoplasmic sperm injection; IVF, in vitro fertilization; NA, not applicable; OR, odds ratio.

^a^ Statistical significance was set at *P* < .05.

To assess the association between infection based on sex with oocyte- or embryo-related outcomes, the study population was stratified into female partners, male partners, and both sexes infected during COS (eTable 1 in Supplement 1). No statistical difference was found in patient and cycle baseline characteristics among the groups. When compared with the SARS-CoV-2–negative group, the mature oocyte rate (in ICSI), 2PN cleavage rate, top-quality embryo rate, blastocyst formation rate, available blastocyst rate, and top-quality blastocyst rate were significantly decreased in the female SARS-CoV-2–positive group. The male SARS-CoV-2–positive group had significantly decreased blastocyst-related rates, including blastocyst formation rate, available blastocyst rate, and top-quality blastocyst rate. The female and male SARS-CoV-2–positive groups both had a significant decrease in blastocyst formation rate. The multiple linear regression analysis revealed that SARS-CoV-2 infection was negatively associated with blastocyst formation rate and top-quality blastocyst rate when adjusted for variables such as age, BMI, infertility duration, and multiple infertility factors (eTable 2 in Supplement 1).

## Discussion

To the best of our knowledge, this is the second report, with the largest sample size, to investigate the association between SARS-CoV-2 infection during COS with oocyte- and embryo-related outcomes in ART cycles. The results of this multicenter, retrospective cohort study demonstrated that SARS-CoV-2 infection during COS was negatively associated with embryo laboratory outcomes.

In general, the top-quality embryo rate, blastocyst formation rate, available blastocyst rate, and top-quality blastocyst rate were significantly decreased in SARS-CoV-2–infected couples compared with uninfected couples. In contrast, Boudry et al^7^ found that the fertilization rate (82%) and the excellent- and good-quality embryo rate in 16 women infected with SARS-CoV-2 were comparable with those in uninfected patients at their clinic. Recently, Chen et al^8^ reported that infection before oocyte retrieval did not have a significant negative association with oocyte and embryo outcomes. This study also compared subgroups (<7-day, 7- to 14-day, and >14-day groups according to the interval from oocyte retrieval to infection) to the control group and showed a decreased number of 2PN zygotes and good-quality embryos in the less than 7-day group.

The exact mechanisms of the effect of SARS-CoV-2 infection on embryo quality remain unclear. It has been reported that both messenger RNA and protein expression of ACE2 were upregulated in human ovulatory follicles after human chorionic gonadotropin injection.^12^ An in vitro experiment in which granulosa and cumulus were cocultured with SARS-CoV-2 RNA showed that human ovarian cells were susceptible to SARS-CoV-2 infection, suggesting a potential adverse effect of SARS-CoV-2 infection on female fertility.^13^ However, in 2021, the absence of the virus in follicular fluid from a SARS-CoV-2–positive woman was reported for the first time.^14^ Similarly, Boudry et al^7^ confirmed this finding and did not detect viral RNA in the follicular fluid and CCs from 16 women who had a positive test result for SARS-CoV-2 RNA within 48 hours before oocyte retrieval.

The association of COVID-19 with male reproduction is even more controversial. Public data sets have reported a high expression of ACE2 and TMPRSS2 in the testis,^1^ whereas an increasing number of studies indicate that ACE2 is highly expressed and there is no TMPRSS2 coexpression in the testis.^15,16^ Some studies^17,18,19,20,21^ have demonstrated the absence of SARS-CoV-2 RNA in semen samples from infected men, whereas other researchers have reported the presence of SARS-CoV-2 in semen at a low percentage. Similarly, the impact of SARS-CoV-2 infection on sperm parameters has been reported in a large number of studies with conflicting results. Our study indicated that male infection was also associated with decreased embryo and blastocyst quality in terms of blastocyst formation rate, available blastocyst rate, and top-quality blastocyst rate. Regrettably, semen quality on the day of oocyte retrieval was not analyzed in the present study. Intracytoplasmic sperm injection is performed when the sperm quality is too poor, and similar outcomes are obtained with IVF.

Besides its effect on gametes, SARS-CoV-2 infection may also have a detrimental effect on the embryo. Published small conditional RNA sequencing data sets showed that ACE2 was expressed in early embryos before the 8-cell stage and the trophectoderm in late blastocysts, whereas the expression of TMPRSS2 began to increase significantly at the late blastocyst stage, resulting in its significant upregulated coexpression only in the trophectoderm of late blastocysts.^22^ Other studies^3,23^ have confirmed that ACE2 and TMPRSS2 were coexpressed in trophectoderm cells of peri-implantation embryos^9^ and in both ectodermal and trophectodermal cells, indicating susceptibility to SARS-CoV-2 infection. However, the presence of SARS-CoV-2 viral RNA in embryos has not been reported.

In addition to the direct effect of viral invasion of cells, oxidative stress^24^ and aberrant systemic inflammation^25^ may be other possible mechanisms by which SARS-CoV-2 affects female reproduction at the molecular level. Reactive oxidative stress affects multiple aspects of reproductive physiologic processes, including oocyte maturation, fertilization, and embryo development.^26,27^ A large amount of proinflammatory cytokines might also interfere with these processes, which may be a possible explanation for why the blastocyst formation rate and top-quality embryo and blastocyst rates were much lower in couples with SARS-CoV-2 infection.

Our study further analyzed the separate associations of infections in female and male populations with oocyte- and embryo-related outcomes. Significantly decreased mature oocyte rate, 2PN cleavage rate, top-quality embryo rate, blastocyst formation rate, available blastocyst rate, and top-quality blastocyst rate were detected in the female SARS-CoV-2–positive group compared with the SARS-CoV-2–negative group. The male SARS-CoV-2–positive group showed impaired embryo evolutive potentiality with decreased blastocyst formation, available blastocyst, and top-quality blastocyst rates. In addition, the both-positive group only had decreased blastocyst formation. These results indicated that SARS-CoV-2 infection in either partner or both partners during COS was negatively associated with embryo development after IVF or ICSI. However, the sample size of the subgroups was small, and the results should be interpreted with caution.

We found that SARS-CoV-2 infection during COS had no association with the ovarian response in terms of number of oocytes retrieval and peak estradiol levels on trigger day. Our results are consistent with limited reports from limited studies published to date.^6,28^

### Strengths and Limitations

The main strength of the present study is the relatively large sample size of couples experiencing fertility issues who were infected with SARS-CoV-2. In addition, we included samples from 7 reproductive centers in 4 provinces across China, with a fixed proportions in the SARS-CoV-2–positive and SARS-CoV-2–negative groups. In addition, the associations of maternal and paternal infection with embryologic variables were compared separately.

Some limitations have been recognized in this study. First, it was a retrospective study, with inherent biases of data collection that were not uniformly generated under the study protocol. Second, the effect of symptom severity on outcomes was not further analyzed, and there were no specific values for viral loads.

## Conclusions

This cohort study indicates that SARS-CoV-2 infection in either or both partners of couples experiencing fertility issues during COS was negatively associated with embryo-related outcomes, including top-quality embryo and blastocyst, blastocyst formation, and available blastocyst rates. These findings suggest that reproductive physicians should pay attention to this group of patients and should give infected couples adequate counseling.

## Notes

Supplement 1. **eTable 1.** Results of Sex-Based Infection of SARS-CoV-2 in Different Groups **eTable 2.** Multivariable Linear Regression Analysis for the Laboratory Outcomes **eTable 3.** The Main Female Infertility Factors Proportion in SARS-CoV-2 Positive and Negative Groups

Supplement 2.
Data Sharing Statement
