# Supplementary material for: Association of SARS-CoV-2 Infection During Controlled Ovarian Stimulation With Oocyte- and Embryo-Related Outcomes
Source: JAMA Netw Open. 2023 Jul 13;6(7):e2323219. doi: 10.1001/jamanetworkopen.2023.23219 (PMC10346123; doi:10.1001/jamanetworkopen.2023.23219)
Supplement: Supplement 1. — eTable 1. Results of Sex-Based Infection of SARS-CoV-2 in Different Groups eTable 2. Multivariable Linear Regression Analysis for the Laboratory Outcomes eTable 3. The Main Female Infertility Factors Proportion in SARS-CoV-2 Positive and Negative Groups [file jamanetwopen-e2323219-s001.pdf]

Supplementary Online Content

Tian F, Li S, Li N, et al. Association of SARS-CoV-2 infection during controlled ovarian stimulation with oocyte- and embryo-related outcomes. *JAMA Netw Open*. 2023;6(7):e2323219. doi:10.1001/jamanetworkopen.2023.23219

eTable 1. Results of Sex-Based Infection of SARS-CoV-2 in Different Groups
eTable 2. Multivariable Linear Regression Analysis for the Laboratory Outcomes
eTable 3. The Main Female Infertility Factors Proportion in SARS-CoV-2 Positive and Negative Groups

This supplementary material has been provided by the authors to give readers additional information about their work.

© 2023 Tian F et al. *JAMA Network Open.*

eTable 1: Results of sex-based infection of SARS-CoV-2 in different groups

**Characteristics / variable**	**Negative (n=450)**	**Female positive (n=33)**	**Male positive (n=28)**	**Both female and male positive (n=74)**	***P-value***
**Baseline characteristics**					
Female age, median (IQR), years	33 (30-37)	32 (29-38)	34.5 (28.0-36.8)	32 (28-37)	*0.772*
BMI, median (IQR), kg/m^2	22.0 (20.3-24.4)	22.9 (20.8-25.0)	22.7 (20.8-24.5)	22.3 (20.6-25.0)	*0.507*
Number of cycles, median (IQR), numbers	1 (1-2)	1 (1-2)	1 (1-1)	1 (1-2)	*0.174*
Infertility durations, median (IQR), years	3.0 (1.0-4.4)	2.0 (1.0-4.3)	1.2 (1.0-2.0)	2.0 (1.0-4.1)	*0.027*
Infertility types, n (%)					*0.484*
Primary	205 (45.6)	15 (45.5)	17 (60.7)	34 (45.9)	
Secondary	245 (54.4)	18 (54.5)	11 (39.3)	40 (54.1)	
Infertility factors, n (%)					*0.785*
Female factor	260 (57.8)	21 (63.6)	14 (50.0)	46 (62.2)	
Male factor	35 (7.8)	1 (3.0)	1 (3.6)	4 (5.4)	
Both female and male factors	138 (30.7)	11 (33.3)	11 (39.3)	21 (28.4)	
Unexplained	17 (3.8)	0 (0.0)	2 (7.1)	3 (4.1)	
COS protocols, n (%)					*0.964*
GnRH-a	131 (29.1)	9 (27.3)	7 (25.0)	20 (27.0)	
GnRH-ant	230 (51.1)	17 (51.5)	12 (42.9)	37 (50.0)	
Others[#]	89 (19.8)	7 (21.2)	9 (32.1)	17 (23.0)	
**Fertility evaluation and COS protocols, median (IQR)**					
Baseline FSH, IU/L	6.7 (5.6-8.5)	6.2 (4.9-7.5)	6.4 (5.4-7.8)	6.9 (5.5-8.2)	*0.393*
AMH, ng/mL	2.5 (1.3-3.9)	2.7 (1.5-4.5)	2.8 (1.3-4.5)	2.4 (1.2-4.5)	*0.872*
AFC, numbers	12 (7-18)	11 (7-19)	12 (7-22)	12 (7-17)	*0.938*
Duration of COS, days	9 (8-11)	9 (8-11)	10 (7-11)	9 (8-11)	*0.986*

© 2023 Tian F et al. *JAMA Network Open.*

Dosage of Gn, (IU)	2093.8 (1500.0-2614.9)	2100.0 (1472.5-2800.0)	1887.5 (1364.1-2540.6)	1856.3 (1500.0-2493.8)	*0.498*
E2 on trigger day, pg/mL	2036.0 (1314.5-3129.0)	1878.0 (1064.7-2986.0)	2323.6 (1292.3-3488.6)	1832.0 (971.9-3000.0)	*0.209*
P on trigger day, ng/mL	0.8 (0.6-1.2)	0.7 (0.5-1.0)	0.9 (0.6-1.5)	0.7 (0.5-1.2)	*0.255*
LH on trigger day, mIU/mL	2.2 (1.2-3.9)	2.2 (1.2-3.8)	2.6 (0.9-3.6)	2.2 (1.0-3.9)	*0.934*
Number of oocytes retrieved, numbers	10 (6-15)	8 (5-14)	11 (6-20)	10 (6-14)	*0.538*
**Laboratory outcomes**					
Fertilization methods, n (%)					*0.387*
IVF	318 (70.7)	29 (87.9)	20 (71.4)	52 (70.3)	
ICSI	113 (25.1)	3 (9.1)	8 (28.6)	18 (24.3)	
(IVF+ICSI)	19 (4.2)	1 (3.0)	0 (0.0)	4 (5.4)	
Mature oocyte rate (ICSI), n/n (%)	981/1228 (79.9)	24/41 (58.5)*	88/108 (81.5)	185/225 (82.2)	
Oocyte degeneration rate (ICSI), n/n (%)	75/981 (7.6)	0/24 (0.0)	4/88 (4.5)	10/185 (5.4)	
2PN rate, n/n (%)					
IVF	2155/3476 (62.0)	184/277 (66.4)	166/255 (65.1)	337/563 (59.9)	
ICSI	735/981 (74.9)	19/24 (79.2)	76/88 (86.4)	131/185 (70.8)	
2PN Cleavage rate, n/n (%)	2990/3059 (97.7)	203/214 (94.9)*	232/242 (95.9)	483/491 (98.4)	
Top-quality embryo rate, n/n (%)	1557/2990 (52.1)	81/203 (39.9)*	116/232 (50.0)	237/483 (49.1)	
Available embryo rate, n/n (%)	2448/2990 (81.9)	156/203 (76.8)	202/232 (87.1)	383/483 (79.3)	
Blastocyst formation rate, n/n (%)	1353/2990 (45.3)	58/203 (28.6)*	59/232 (25.4)*	189/483 (39.1)*	
Available blastocyst rate, n/n (%)	1070/2990 (35.8)	45/203 (22.2)*	52/232 (22.4)*	162/483 (33.5)	
Top-quality blastocyst rate, n/n (%)	343/2990 (11.5)	13/203 (6.4)*	8/232 (3.4)*	44/483 (9.1)	

[#] Others, including mild stimulation and luteal-phase stimulation protocols.

Compared with SARS-CoV-2 negative group respectively: *, *P<0.01.*

© 2023 Tian F et al. *JAMA Network Open.*

eTable 2: multivariable linear regression analysis for the laboratory outcomes

	Top-quality embryo/2PN cleavage				Blastocyst formation/2PN cleavage				Available blastocyst/2PN cleavage				Top-quality blastocyst/2PN cleavage			
Variables	B	Beta	P	95% CI of B	B	Beta	P	95% CI of B	B	Beta	P	95% CI of B	B	Beta	P	95% CI of B
(Constant)	36.69		0.01	8.08 to 65.35	43.90		0.00	18.57 to 69.23	44.83		0.00	21.44 to 68.22	11.15		0.08	-1.15 to 23.46
Age	0.17	0.03	0.59	-0.46 to 0.81	-0.42	-0.07	0.14	-0.99 to 0.14	-0.46	-0.08	0.08	-0.98 to 0.06	-0.04	-0.02	0.76	-0.32 to 0.23
BMI	0.38	0.04	0.41	-0.54 to 1.30	-0.66	-0.07	0.11	-1.48 to 0.15	-0.81	-0.09	0.03	-1.56 to -0.06	-0.35	-0.08	0.08	-0.75 to 0.04
Infertility durations	0.22	0.02	0.63	-0.69 to 1.12	-0.01	0.00	0.98	-0.81 to 0.79	-0.03	0.00	0.94	-0.77 to 0.71	0.00	0.00	0.99	-0.39 to 0.39
Infertility factor (anovulation)*	0.84	0.01	0.90	-12.22 to 13.90	-8.85	-0.06	0.13	-20.39 to 2.70	-4.64	-0.04	0.39	-15.30 to 6.03	-3.40	-0.05	0.23	-9.00 to 2.21
Infertility factor (endometriosis)*	7.29	0.03	0.45	-11.65 to 26.23	0.61	0.00	0.94	-16.13 to 17.36	1.45	0.01	0.85	-14.02 to 16.92	-1.07	-0.01	0.80	-9.20 to 7.07
Infertility factor (DOR)*	6.37	0.05	0.25	-4.47 to 17.22	-2.81	-0.02	0.56	-12.40 to 6.77	0.11	0.00	0.98	-8.75 to 8.96	-3.16	-0.06	0.18	-7.82 to 1.49
Group (positive)#	-3.90	-0.05	0.21	-10.02 to 2.22	-8.25	-0.12	<0.01	-13.67 to -2.84	-4.28	-0.07	0.09	-9.28 to 0.71	-3.10	-0.09	0.02	-5.73 to -0.47

* Tubal factor was set as the reference category.

# SARS-CoV-2 negative group was set as reference category.

© 2023 Tian F et al. *JAMA Network Open.*

eTable 3: The main female infertility factors proportion in SARS-CoV-2 positive and negative groups

**Female factors (n=341)**	**SARS-CoV-2 negative (n=260)**	**SARS-CoV-2 positive (n=81)**	***P value****
Tubal factor, n (%)	190 (73.1)	58 (71.6)	*0.78*
Anovulation, n (%)	35 (13.5)	11 (17.3)	*1.00*
Endometriosis, n (%)	14 (5.3)	6 (7.4)	*0.59*
DOR, n (%)	63 (24.2)	23 (28.4)	*0.47*

Note: DOR, diminished ovarian reserve.

* Statistical significance was set at $P < 0.05$ in the Chi-squared tests.

© 2023 Tian F et al. *JAMA Network Open.*
