# Supplementary material for: Association of SARS-CoV-2 Infection During Controlled Ovarian Stimulation With Oocyte- and Embryo-Related Outcomes
Source: JAMA Netw Open. 2023 Jul 13;6(7):e2323219. doi: 10.1001/jamanetworkopen.2023.23219 (PMC10346123; doi:10.1001/jamanetworkopen.2023.23219)
Supplement: Supplement 2. — Data Sharing Statement [file jamanetwopen-e2323219-s002.pdf]

Data Sharing Statement

Tian. Association of SARS-CoV-2 Infection During Controlled Ovarian Stimulation With Oocyte- and Embryo-Related Outcomes. *JAMA Netw Open*. Published July 13, 2023.
doi:10.1001/jamanetworkopen.2023.23219

Data
Data available: No
